# Blackwater Fever Treated with Steroids in Nonimmune Patient, Italy

**DOI:** 10.3201/eid2904.221267

**Published:** 2023-04

**Authors:** Anna Rita Di Biase, Dora Buonfrate, Francesca Stefanelli, Giorgio Zavarise, Erica Franceschini, Cristina Mussini, Lorenzo Iughetti, Federico Gobbi

**Affiliations:** University of Modena and Reggio Emilia, Modena, Italy (A.R. Di Biase, F. Stefanelli, C. Mussini, L. Iughetti);; IRCCS Sacro Cuore don Calabria Hospital, Negrar, Verona, Italy (D. Buonfrate, G. Zavarise, F. Gobbi);; Azienda Ospedaliero-Universitaria of Modena, Modena (E. Franceschini)

**Keywords:** Blackwater fever, malaria, hemoglobinuria, Plasmodium falciparum, quinine, artemisinin derivatives, parasites, Italy

## Abstract

Causes of blackwater fever, a complication of malaria treatment, are not completely clear, and immune mechanisms might be involved. Clinical management is not standardized. We describe an episode of blackwater fever in a nonimmune 12-year-old girl in Italy who was treated with steroids, resulting in a rapid clinical resolution.

On May 9, 2022 (day 1), a 12-year-old girl was admitted to the pediatric emergency department of Modena Hospital, Modena, Italy, for persistent fever (>2 days) and lethargy. She had returned to Italy from a family excursion to Nigeria 11 days prior to admission. She did not take malaria prophylaxis. Her initial hospital evaluations revealed severe thrombocytopenia, increased total bilirubin, and lactate dehydrogenase (Table). Hemoglobin was within normal range. A rapid diagnostic test for malaria was positive, and blood smears confirmed high *Plasmodium falciparum* parasitemia (26%).

**Table:** Laboratory exams during hospitalization for a 12-year-old girl with blackwater fever, Modena, Italy*

Parameter	Day 1	Day 2	Day 4	Day 7	Day 10	Day 13	Day 23	Follow-up
Labwork								
Hemoglobin, g/dL	12.3	9.1	7.8	10.2	7.9	9.9	10.1	12.6
Platelets, 10^3^/mmc	27	45	96	79	354	379	451	301
Bilirubin, mg/dL	1.96	1.53	1.21	1.06	1.57	2.25	0.61	0.42
ALT, U/L	77	79	389	446	260	124	62	12
AST, U/L	NA	NA	1398	1435	254	128	35	NA
LDH, U/L	1870	1732	7676	7200	5147	5646	1715	513
PCR, mg/dL	23.8	15.2	24.3	11.6	1.5	1.8	<0.2	<0.2
Hemoglobinuria	N	N	Y	Y	Y	Y	N	N
Treatment								
Artesunate	×	×	–	–	–	–	–	–
Artemether/lumefantrine	–	–	×†	–	–	–	–	–
Blood transfusion	–	–	×	–	×‡	–	–	–
Steroids	–	–	–	–	–	×§	–	–

*ALT, alanine aminotransferase; AST, aspartate aminotransferase; LDH, lactate dehydrogenase; N, no; NA, not applicable; PCR, polymerase chain reaction; Y, yes; ×, administered; –, not administered. †Days 3–5. ‡Days 10, 12 and 16. §Days 13–17.

The girl was admitted to the intensive care unit, where she received 4 doses of 2.4 mg/kg intravenous artesunate. Therapy was then switched to artemether/lumefantrine (80/480 mg dose, administered orally in 6 doses). Blood smears were negative for *P. falciparum* starting on day 4. Because the girl’s hemoglobin levels had dropped steadily from the time of admission (Table), she received a blood transfusion on day 4. On day 5, she was discharged from the intensive care unit in good clinical condition and moved to the pediatric ward, where hyperchromic urine samples were observed. Empiric antibiotic treatment was started (ceftriaxone first, then piperacillin/tazobactam) to treat her persistent fever. Result of blood and urine cultures were negative, as were investigations for SARS-CoV-2 and other respiratory viruses. Chest radiographs and brain computed tomography scans (the latter performed to investigate additional causes of lethargy) had unremarkable results. Ultrasound examination revealed biliary sludge. Because the patient’s hemoglobin level continued to drop, she received additional blood transfusions on days 10 and 12.

Our team suspected blackwater fever (BWF), a complication of *P. falciparum* infection, and colleagues from a referral center for tropical diseases confirmed the diagnosis and recommended administration of steroids. We prescribed a 5-day treatment course of oral prednisone (1.3 mg/kg), starting on day 13. We tapered off the dose over the next 15 days and administered another blood transfusion on day 16. Symptoms cleared completely the day after steroid treatment began, and urine samples became normochromic 7 days later. The patient was discharged in good clinical condition on Day 23. One month later, blood test results were unremarkable (Table). No abnormal hemoglobin or glucose-6-phosphate-dehydrogenase results were noted.

BWF is a condition characterized by massive hemolysis after treatment for acute malaria, with clinical symptoms that include hemoglobinuria, anemia, jaundice, and fever (*1*–*3*). The name of the syndrome relates to the presence of dark urine noted in affected patients. An apparent decrease in cases of BWF was noted when artemisinin compounds replaced quinine as first-line treatment for malaria (*3*,*4*). However, a randomized, controlled trial comparing quinine versus artesunate for treatment of severe malaria in children and found frequency of BWF did not differ significantly between the 2 study arms (p = 0.076) (*5*).

Artesunate has been associated with hemolytic anemia, a condition that differs from BWF in that hemolysis is delayed (usually starting from 2 to 3 weeks following initiation of artesunate therapy, although some cases can occur earlier); most important, hemoglobinuria is not reported. Another differentiating factor between the 2 conditions is that hemolysis due to artesunate is extravascular and, in BWF, hemolysis is intravascular (*6*).

The pathophysiologic mechanisms causing BWF are not completely understood, and no definitive evidence has emerged from investigations into the role of antimalarial drugs (mostly quinine, with some reports about halofantrine and mefloquine), characteristics of the human host (e.g., glucose-6-phosphate-dehydrogenase deficiency), and parasite type (e.g., different *Plasmodium* strains) (*1*,*7*,*8*). No relationship has been reported between high levels of parasitemia and development of BWF. Because most cases of BWF arise in nonimmune persons, immune mechanisms have been suspected to cause the hemolysis (*8*). Nevertheless, many cases have been observed in children >5 years of age who resided in malaria-endemic areas and were suspected to carry at least partial immunity against *Plasmodium* spp. (*8*). Studies suggest that those children failed to attain protective immunity against malaria and, indeed, showed an immune profile similar to expatriates in Europe. Different mechanisms have been speculated to be involved in activating the immune response leading to BWF, including excessive complement activation and presence of a malaria immune complex antigen-antibody (*8*).

Treatment with steroids, as was determined for this patient, has been previously instituted in a malaria-endemic setting (*9*). Although evidence is not sufficient to recommend this therapeutic approach, it seems reasonable from a pathophysiologic standpoint and deserves further evaluation. Besides steroids, the 2 pillars of BWF management are blood transfusion and refraining from drugs possibly causing the syndrome (*9*). Because of the rarity (and neglect) of BWF (*3*), randomized controlled trials comparing treatment options are not currently feasible. Nonetheless, the disease is a serious consequence for children who are susceptible; there is a 3-fold higher risk of death for children with severe anemia and BWF than for children with severe anemia and no BWF (*2*). Considering that statistic and the poor outcomes observed in the 6 months following a BWF episode (*2*), evidence in support of clinical management is clearly needed.
